# Attraction to conspecific social-calls in a migratory, solitary, foliage-roosting bat (*Lasiurus cinereus*)

**DOI:** 10.1038/s41598-022-13645-9

**Published:** 2022-06-09

**Authors:** Gabriel A. Reyes, Joseph M. Szewczak

**Affiliations:** 1 Department of Biological Sciences, Cal Poly Humboldt, Arcata, CA 95521 USA; 2 Present Address: Dixon Field Station, Western Ecological Research Center, U.S. Geological Survey, Dixon, California 95620 USA; 3 Present Address: Department of Environmental Science and Policy, University of California, Davis, California 95616 USA

**Keywords:** Animal migration, Behavioural ecology

## Abstract

As a migratory, cryptic, foliage-roosting bat with a mostly solitary roosting behavior we have an incomplete understanding of the social behavior of the hoary bat, *Lasiurus cinereus*. In this species most social interactions between conspecifics are thought to involve mating behavior or territorial disputes. Developing a more complete understanding of the social behavior of this species would provide critical insight to address conservation challenges including high fatality rates from wind turbines during the period of fall migration. We tested the response of hoary bats to conspecific social call playback during the spring and fall migration to: (1) test whether conspecific social call broadcasting attracts or repels individual bats; (2) examine whether there are seasonal differences in these responses; (3) describe the structure and variation of recorded social calls; and (4) test whether conspecific social call playback can increase capture success. Hoary bats were attracted to social call broadcasting during both the spring and fall migration. Hoary bats produced social calls during the spring and fall migration, and when only males were present, suggesting a social function not associated with mating. While calls were variable in frequency and length, social calls tended to be a consistent upsloping shape. Attraction to social calls suggests social interactions not associated with mating behavior in hoary bats, and this technique proved successful as an acoustic lure to aid in capture and study of this elusive species.

## Introduction

Social behavior is an important component of natural history which impacts management, monitoring, study methods, and conservation of wildlife^1^. Knowledge of social behavior can influence our understanding of demography, minimum population viability, biogeographic patterns, and gene flow and dispersal^2^. Importantly, a lack of knowledge about social behavior can result in failures of conservation strategies and inefficient use of resources^2^. Communication among conspecifics is an important component of social behavior, and can include passive or active signals using a variety of senses (e.g., sight, touch, scent, and sound), with signals sometimes leading to approach (e.g., a pup attracting a parent) or repel behaviors (e.g., territorial defense), or for conveyance of information (e.g., alarm calls), depending on context and relationship between individuals^3^.

Studying social behavior of cryptic animals presents challenges to researchers, in that communication and interactions between conspecifics are often not readily observable. Call broadcasting is often used to study social behavior of cryptic species^4^, and can be utilized to increase sampling or detection efficiency^5,6^. Broadcasting conspecific or sympatric vocalizations has provided an effective way to increase detection or capture probability in some species of bats. For example, broadcast of social call recordings can serve as a lure to increase capture rates of the elusive and threatened bat *Myotis bechsteinii* in netting locations intentionally removed from flyways^7^. Many researchers have reported anecdotes of increased capture rates of many species following the capture of a bat emitting distress calls while tangled in a mist net or when handled nearby or through distress call broadcast^8^. In addition, bats eavesdrop on echolocation calls of other bats and gain information about the environment beyond the range of their own echolocation calls^9–11^.

The nocturnal migratory behavior of the hoary bat (*Lasiurus cinereus*), coupled with its preference for solitary roosting high in the foliage of trees^12^ has hampered efforts to study their seasonal movements and social behavior. There has been a resurgence of interest in hoary bats in North America due to a high number of fatalities suffered at wind energy developments (about 40% of all fatalities documented at wind turbines in North America)^13,14^, which are likely to lead to population declines^15,16^. The highest rates of bat fatalities at wind turbine sites coincide with late summer and early fall, corresponding with the presumed period when hoary bats mate, as do other migratory species being impacted by wind turbines, including the eastern red bat (*Lasiurus borealis*) and the silver-haired bat (*Lasionycteris noctivagans*)^13,17–19^. The coincidence between mating behavior and fatalities at wind farms has led some researchers to hypothesize that seasonally variable social factors play a role in these fatalities^20–22^. Because so little is known about the behavior of hoary bats during migration, understanding how individuals react to conspecific social calls could improve understanding of this species’ natural history to support its conservation and management.

In this study we aimed to: (1) test whether broadcasting conspecific social-calls would attract or repel hoary bats, (2) examine whether there are seasonal differences in these responses, and (3) investigate and quantify any variation and structure of recorded social calls, and (4) test whether conspecific social call playback can increase capture success.

## Results

We completed 104 half-hour social call and control (silence) playback trials, 56 in the spring, and 48 in the fall (Table 1). We captured a total of 31 hoary bats at the experimental net, 30 during social call broadcast and 1 during control broadcast (Table 1). Mean number of captures per half-hour period for each broadcast condition was: zero for control during the fall and 0.036 for spring (0.019 overall); 0.333 for Laso1 (*L. cinereus* social call file 1, see “Methods” section) during the fall and 0.571 during the spring (0.462 overall); and 0.714 for Laso2 (*L. cinereus* social call file 2, see “Methods” section) during the fall and 0.667 during the spring (0.692 overall).

**Table 1:** Number of hoary bats (*Lasiurus cinereus*) captured during different conspecific social call playback treatments and control during spring and fall (number of trials in parentheses).

Treatment	Spring	Fall	Total
Control	1 (28)	0 (24)	1 (52)
Laso1	8 (14)	4 (12)	12 (26)
Laso2	10 (14)	8 (12)	18 (26)

**Figure 1:**
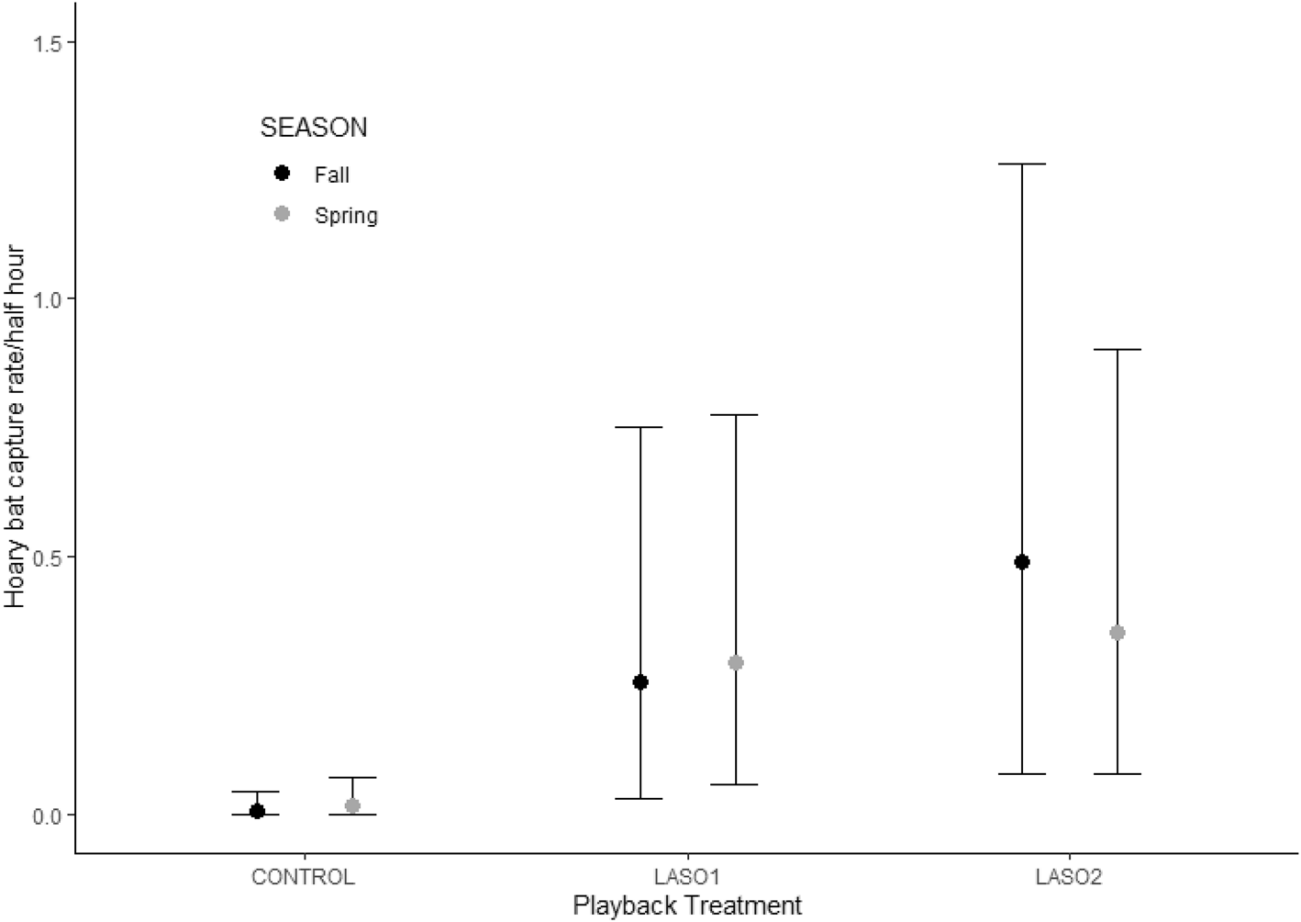
Model result estimates of capture rate of hoary bats (*Lasiurus cinereus*) per half hour during control (n = 52), and social call broadcasting Laso1 (n = 26) and Laso2 (n = 26), with 95% credible intervals, from posterior of Treatment and Season interaction.

Humidity and season were highly correlated (r =  − 0.9) so we removed humidity from all subsequent analysis. Once Humidity was removed all variance inflation factor values were under 3. For one fall night with missing acoustic activity data we used the average value for fall. Playback treatment for both Laso1 and Laso2 had a strong positive effect on capture rate over control conditions. All other parameters had fairly weak effects on capture rate, with seasonal interaction effects indicating a slightly higher capture rate during the spring (Table 2). The modeled results of our multi-level model (Poisson distribution, Bayesian framework) estimate mean hoary bat capture rates per half hour treatment of 0.018 (0–0.076), 0.324 (0.068–0.826), and 0.395 (0.093–0.972) for control, Laso1, and Laso2 during Spring, and 0.006 (0–0.048), 0.15 (0.008–0.529), and 0.368 (0.042–1.014) for control, Laso1, and Laso2 during Fall (Fig. 1).

**Table 2:** Parameter estimates, prior designations, estimated error, and 95% credible intervals for multilevel model examining response of hoary bats (*Lasiurus cinereus*) to social call broadcasting.

Model term	Prior	Estimate	Est. error	95% lower CI	95% upper CI
Intercept (Control, Fall)	Normal (0, 10)	− 7.23	2.75	− 13.55	− 3.10
Laso1, fall	Normal (0,10)	5.60	2.73	1.42	11.93
Laso2, fall	Normal (0,10)	6.31	2.71	2.26	12.56
Control, spring	Normal (0,10)	2.36	2.96	− 2.75	8.87
LACI acoustic activity	Normal (0,10)	0.22	0.39	− 0.51	1.06
Temperature	Normal (0,10)	0.16	0.26	− 0.34	0.68
Moon phase	Normal (0,10)	− 0.29	0.31	− 0.91	0.34
Wind speed	Normal (0,10)	0.17	0.24	− 0.30	0.64
Sky	Normal (0,10)	− 1.03	0.67	− 2.55	0.06
Laso1*spring	Normal (0,10)	− 2.14	2.93	− 8.56	2.93
Laso2*spring	Normal (0,10)	− 2.66	2.90	− 9.05	2.28
Night (random)	Cauchy (0, 2)	0.97	0.51	0.14	2.17

### Social call characteristics

**Figure 2:**
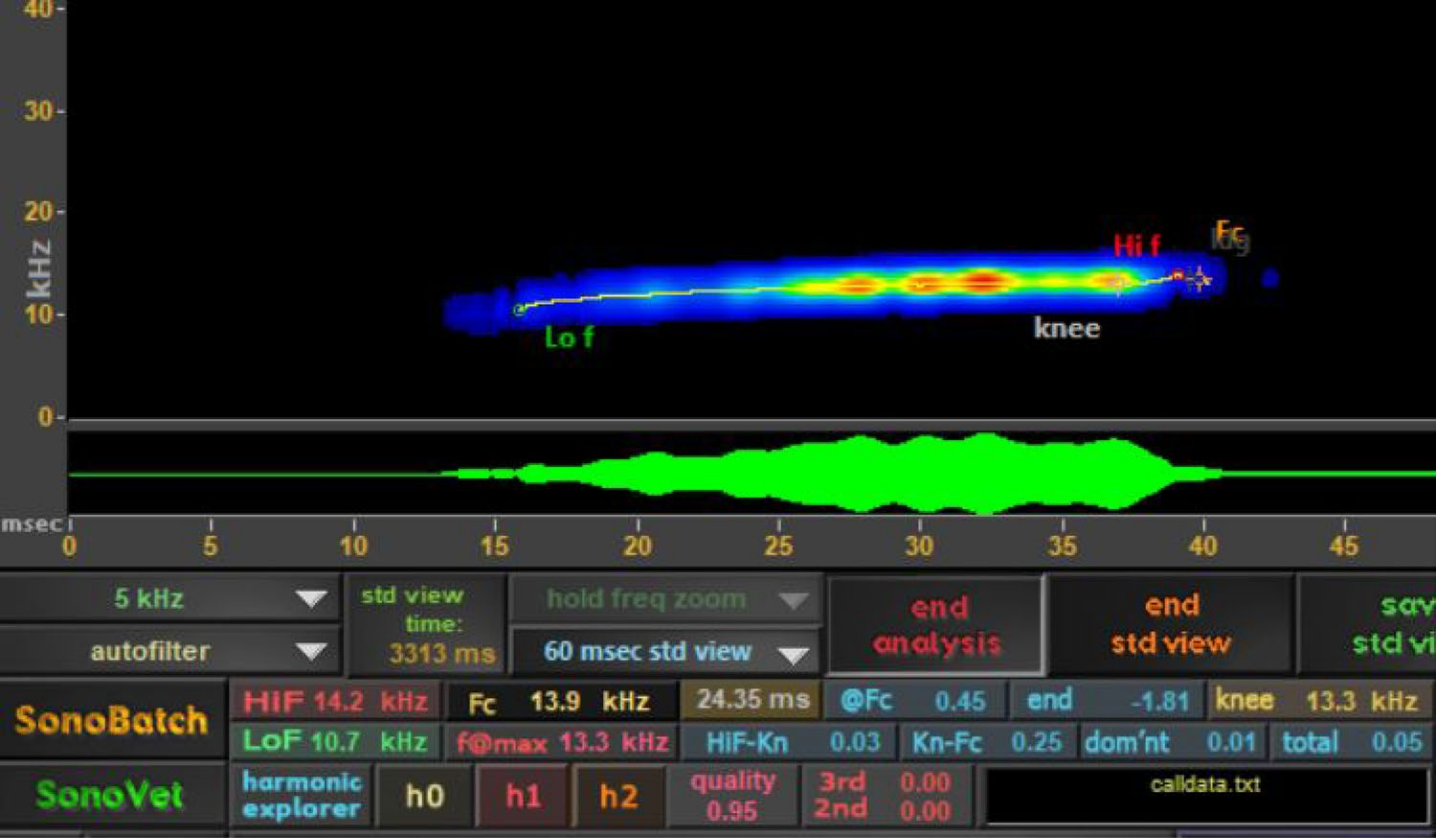
Typical hoary bat (*Lasiurus cinereus*) social call, showing upsloping call shape, and parameters used to describe calls displayed in SonoBat. Time in milliseconds is on the x axis, and frequency in KHz on the Y axis, displayed in SonoBat.

We calculated descriptive parameters for 263 hoary bat social calls from 98 files recorded in New Mexico. Despite observers often hearing hoary bat social calls during the fall migration, the detectors did not record any social calls during the fall migration. We considered calls to be social calls when they were not part of a regular search phase or terminal sequence of calls^23^, and when they sloped up or were unlike typical echolocation calls known from hoary bats and were mostly audible to human hearing. While social calls were variable, they mostly were a variation of an upsloping low-frequency call (Fig. 2). Each call file contained between 1 and 10 social calls, with an average of 2.71 social calls per file (SD = 1.71). The average start and end frequencies were 13.08 kHz and 15.1 kHz with an average duration of 33.25 ms, and the average frequency with maximum power was 14.8 kHz (Table 3).

**Table 3:** Mean (standard deviation in parenthesis), minimum, and maximum social call parameters from 98 recorded call files containing 263 hoary bat (*Lasiurus cinereus*) social calls.

Variable	Average	Min	Max
Start frequency (kHz)	13.08 (2.46)	7.96	20.37
End frequency (kHz)	15.1 (1.7)	8.39	21.59
Bandwidth (kHz)	2.84 (1.59)	0	8.09
Call duration (ms)	33.25 (12.42)	11.75	99.78
Fmax (kHz)	14.8 (1.51)	8.31	18.81

## Discussion

Broadcasted social calls attracted hoary bats during both the spring and fall migration. Broadcasting conspecific social calls increased hoary bat capture rates at netting sites intentionally removed from normal capture locations. We had very low capture rates during control periods, because we intentionally placed nets in locations removed from flyways to reduce incidental captures. Moreover, capture rates of hoary bats tend to be low even in many locations where they are known to occur^24,25^, and capture rates of approximately one bat per hour in a single mist net suggest a very strong attraction response to broadcasted calls.

Hoary bat activity, as measured by acoustic monitoring was not associated with increased capture rates in response to call broadcasting. However, subsequent research has shown that hoary bats periodically use higher frequency, inconspicuous calls, or do not constantly echolocate during the fall, which may mean acoustic monitoring did not effectively measure hoary bat activity in the vicinity of our trials^26,27^. We recorded substantially higher acoustic activity during the spring migration, which could represent either more hoary bats and/or bat activity, or a seasonal difference in echolocation or flight behavior such as differences in flight altitude^27^. It remains unknown if hoary bats use inconspicuous calls or fly in silence during spring migration or other times of year other than the fall when these inconspicuous echolocation behaviors were observed, and seasonally variable behavior could affect detectability or exposure to our playback trials in ways not captured by our acoustic activity covariate. In addition, while we did audibly hear social calls of hoary bats during the fall, we did not record any during fieldwork for this study, which may be an artifact or due to differences in social behavior, context, or number of hoary bats present in the area during our trials.

We only captured one female during trials in New Mexico, and were unable to locate any females during the fall migration in coastal regions of California, despite high concentrations of males in the area during what is presumably the mating season. In New Mexico, during spring migration, females migrate through the study area before males^28^, with very little temporal overlap. As a result, we were unable to determine sex specific responses to call playback, however we have subsequently captured several female hoary bats and Ope’ape’a (Hawaiian hoary bat, *L. semotus*) using call playback during capture and radio-tracking studies (GAR, pers. obs.).

It is difficult to elucidate the meaning of social calls based on the behaviors observed in the field. In bats, social call complexity often reflects social behavior complexity, with a range of uses including but not limited to attracting mates, locating pups within colonies, defending roosting or foraging territory, and attracting bats to roosts^10^. Attraction to conspecific call broadcasting could indicate positive social interactions (e.g., maintaining group cohesion or investigation) or agonistic behavior (e.g., hoary bats approaching to chase conspecific bats), as has been observed in other bat species^29^ and in hoary bats during the maternity season^30^. We did not observe any obvious instances of aggressive hoary bat interactions, and the social calls differ from hisses and clicks that hoary bats use defensively (Fig. 2). We would also audibly hear pairs of hoary bats calling in close proximity to each other, with no indication of aggressive or territorial responses, and these calls being low frequency and audible to humans means that they attenuate at greater distances than hoary bat echolocation calls.

Aggressive or territorial interactions in many taxa are often driven by seasonally variable contexts, such as mating, defending food resources, or rearing of young. It may be unlikely that migrating hoary bats would expend energy defending territory during migration when they are utilizing roosts or foraging habitat for such limited periods of time (i.e., a few hours to a day). During active migration birds are often not territorial even when foraging at stopover sites^31^, and there may be benefits to maintaining group cohesion during migration including navigation and identification of favorable habitat. It is unknown if hoary bats utilize stopover sites for refueling during migration. However the silver-haired bat *Lasionycteris noctivagans* was found to utilize a migration stopover site in Long Point, Canada, where they opportunistically foraged for short periods of time (1 to 2 days^32^). Tracking studies would be required to determine temporal patterns of site usage by individual bats to examine stopover behavior.

As we had recorded most of our initial social calls during late summer and early fall when hoary bats mate^21^, we had originally hypothesized that these social calls were associated with mating behavior, which would have been consistent with observations in this study had we found both increased attraction during the fall, and less attraction to calls during the spring. However, social calls attracted hoary bats effectively during both the spring and fall migration. In addition, from acoustic recordings and capture observations in the field, hoary bats produced many social calls during the spring migration when only males were present. There is a possibility, due to our lack of understanding of the mating systems of hoary bats that some mating may continue into the spring. However the majority of taxonomic, physiological, and observational data suggests mating behavior ends by the spring migration^19,33^, and the majority of females are already pregnant when travelling through New Mexico^28^. While hoary bats may or may not use social calls as a component of mating behavior, social calls recorded during the spring likely serve purposes not associated with mating.

Previous studies describe the hoary bat as solitary throughout most of the year, which would imply only brief social interactions limited to mating or association with offspring, and the many historical accounts of aggregations of hoary bats are thought to be related to mating behavior^20,33,34^. However the use of, and attraction to, social calls during both spring and fall migration supports that these calls are used for social interactions beyond mating behavior. Further research may determine if hoary bats use these social calls to maintain group cohesion during migration, and what, if any, relationships exist between individual hoary bats that appear to be migrating together. Baerwald and Barclay^35^ found that geographic and genetic relationships of hoary bats and silver-haired bat carcasses collected at wind turbines were not more closely related than expected by chance, which provides some evidence that groups of migrating hoary bats may not form based on kinship.

Many studies hoping to elucidate the causes of fatalities at wind energy facilities have focused only on the fall migration period when bats are most often killed^13,20,36^. However hoary bats migrate during the spring as well, when they do not suffer high fatality rates. Investigating the spring migration presents a valuable baseline to compare behavioral changes and other factors that may place hoary bats or other impacted species at risk. If social behavior makes a major contribution to the risk of fatalities at wind energy developments, then social behavior should differ between spring and fall migration. We did not find a large difference in response to social calls between seasons. While this represents just an initial study into the social calling behavior of hoary bats during migration, it provides some conclusions to guide subsequent investigations: (1) detecting hoary bat social calls does not necessarily indicate mating behavior, and (2) researchers should be cautious in interpreting evidence of social interactions during the fall at wind energy sites as evidence of mating behavior as in the mating landmarks hypothesis^22,37^. Because it can separate out mating from other behavioral components, comparing spring and fall migration can benefit the investigation of social and other behaviors in hoary bats and other migratory species. Comparing flight behavior, diet, roost selection, hormonal and physiological changes, and further studies of social interactions including scent and, between the spring and fall migration will allow researchers to elucidate which behaviors change seasonally and which may underlie seasonal patterns of wind turbine fatalities. Additionally, exploring social attraction to audible sounds produced by turbines or other potential signals that could seasonally elicit social attraction could lead to additional insights.

Hoary bats have proven challenging to capture and study in many locations across their range^24^, driven by their solitary tree roosting behavior and as they often fly out of the reach of mist nets or ground-based acoustic monitoring stations^36,38^. Using call broadcasting to increase capture rates can be a useful research tool, especially in locations where the habitat does not provide any ideal capture locations. Using this technique we have captured hoary bats on coastal sand dunes, in large open fields, and in groves of Eucalyptus trees adjacent to wind energy sites, all of which would normally yield low bat capture success without the use of lures. The ability to capture hoary bats more reliably is a great asset for research and conservation throughout the range of hoary bats.

Our study tested the use of social call playback as a methodology to study the social behavior of hoary bats during migration, and the utility of using call playback as a research tool and acoustic lure for hoary bats. Increasing capture rates from conspecific social call playback during mating and non-mating season indicates social interactions during both migratory periods, despite the solitary roosting behavior of this species. Future studies to elucidate the behavioral function of these calls, and response during non-migratory seasons could refine our understanding of social behaviors of this elusive bat species.

## Methods

### Study area

We conducted fieldwork at locations where we could expect high concentrations of hoary bats to occur during their spring and fall migrations: from 15 May to 15 June 2010 in New Mexico, primarily in Sandoval and Bernalillo Counties, and from 31 August to 17 November 2010 at sites in Humboldt and Marin counties in Northern California. Sites were generally associated with ponds or riparian corridors, and several were selected based on historic capture records.

### Capture and call playback

We used mist net capture rate to measure hoary bat responses to social call broadcasting^39^. To minimize incidental capture of hoary bats, we did not place mist nets across flyways as conventional in North America bat capture surveys. Instead, we positioned a 9-m mist net in a location selected to minimize incidental captures, such as against vegetation, or under an overhanging canopy of a tree. We avoided areas of dense vegetation and more often placed nets either parallel to flyways, on the edge of an open field, or under a tree in a field. We used the AT800 Ultrasonic Transmitter (Binary Acoustic Technologies, Tucson, Arizona) to broadcast social calls. The AT800 is an omnidirectional speaker system originally designed to broadcast ultrasonic noise to deter bats from flying around wind turbines, however it can be used to play bat calls as well. The AT800 speaker was placed on a tripod approximately 1.5 m off the ground and about 0.3 m behind the net, typically hidden against vegetation or a tree trunk.

**Figure 3:**
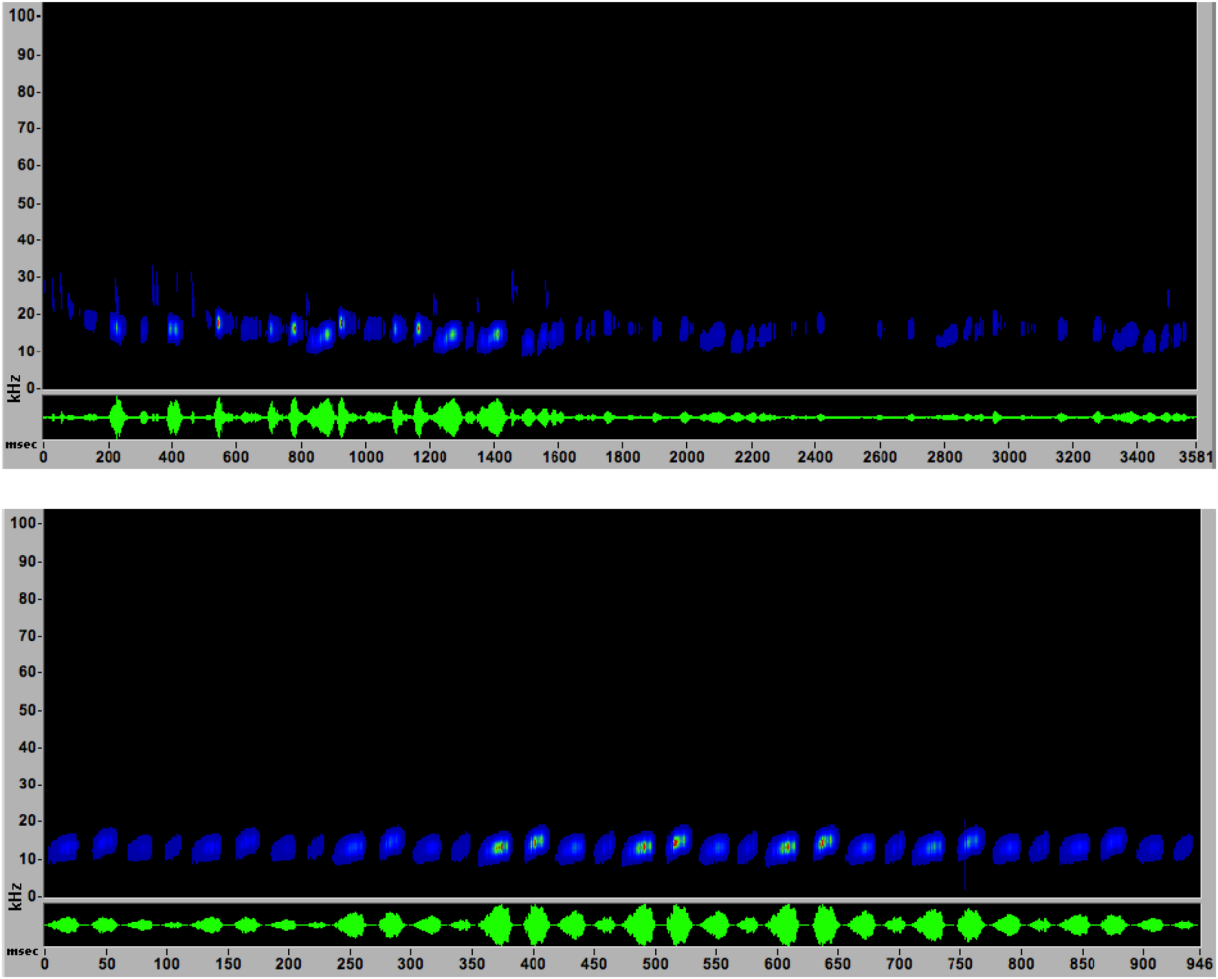
Hoary bat (*Lasiurus cinereus*) social playback files Laso1 (**a**) and Laso2 (**b**), constructed from wild recorded social calls recorded in New Mexico, USA, used in broadcast trials. Laso1 contained an assortment of social call files strung together, while Laso2 contained a sequence of 4 calls from one recording, looped to create a longer sequence. Time in milliseconds is on the x axis, and frequency in KHz on the Y axis, displayed in SonoBat.

Social call files were recorded from in-flight .wav call files recorded primarily on Pettersson D240x bat detectors in field conditions. Social call broadcast files were created in Audacity (Audacity Team 2012, Audacity^®^). We used 2 social call files for broadcasting: Laso1 and Laso2 (Laso for *Lasiurus* social call). Laso1 consisted of three hoary bat passes recorded in July and August, between 2006 and 2008, by other researchers during acoustic surveys in Montana, strung together and looped to create a sequence of 44 social calls with some interspersed echolocation calls totaling 15 s in length (Fig. 3a). Laso2 consisted of one sequence of four social calls we recorded during spring fieldwork in New Mexico, looped to create a sequence of 32 social calls, followed by silence, totaling 15 s in length (Fig. 3b). We created the control file by sampling interpulse noise from the Laso1 broadcast file and looping it, so during control periods the speaker would be operating by broadcasting “silence”. Because hoary bat social calls are audible, we were aware of treatment condition, but the unambiguous response variable of bat capture unlikely subjected the results to observer bias.

Broadcast trials began at least half an hour after sunset. We used coin flips to select whether to begin each trial with the control or treatment playback file, which played on repeat for a half hour. Each subsequent half hour we alternated between treatment levels and control (e.g., Control, Laso1, Control, Laso2). At the start of each half hour trial we recorded temperature (°C), percent relative humidity, Beaufort wind speed score (calm, light breeze, light wind, windy), and sky score (clear, partly cloudy, cloudy, rain). Trials were stopped in the event of rain. We obtained percent moon illumination from the online moon phase calculator of the U.S. Naval Observatory, Astrological Applications Department (http://aa.usno.navy.mil/data/docs/RS_OneDay.html). We monitored the net at least every 15 min during trials. For captured bats we recorded: species, sex, juvenile or adult (using ossification of the carpals^40^), reproductive status (null parous, descended, pregnant, lactating, or post-lactating^41^), forearm length (mm), and mass (grams). We were able to release most bats within one half an hour of capture.

All experimental procedures, bat capture, and handling were performed in accordance with relevant guidelines and were approved Humboldt State University Institutional Animal Care and Use Committee (IACUC Protocol 09/10.B.32-A). Fieldwork was conducted under permits from the California Department of Fish and Game (SC-010919), New Mexico Department of Game and Fish (Permit Number 3466), and relevant land management agencies.

In addition to mist netting, we conducted acoustic monitoring during trials to examine hoary bat activity and to record social vocalizations of hoary bats. We used a Pettersson D240x time expansion bat detector, set to record for 1.7 s, with a division ratio of 10, connected to either an iRiver IFP-895 (iRiver America, Irvine, California) or a Zoom H2 (Zoom North America, Ronkonkoma, New York) to record .wav files. We classified calls to species using SonoBat 3.0 West (SonoBat, Arcata, CA). However, as the program did not automatically recognize social calls, we manually searched through all recorded call files to identify hoary bat social calls.

To describe social calls we used SonoBat to extract the following parameters: start frequency; end frequency; duration; bandwidth; maximum frequency (Fig. 2). We calculated the mean and standard deviation for social call parameters.

### Statistical analysis

To examine the effect of conspecific social call broadcast on hoary bat capture rates, we used a multi-level model with a Poisson distribution in a Bayesian framework. We excluded nights where no hoary bats were detected by any observation method (acoustic monitoring, capture in mistnets, audible or visual observations). We used the number of hoary bats captured in each half hour period as the response variable, in response to treatment (Control, Laso1, Laso2), with an interaction with Season (spring or fall), and additive variables of nightly acoustic activity of hoary bats (hoary bat passes/hour), temperature (°C), percent humidity, wind speed (ordinal, 0 through 3), and percent moon illumination. We included sampling night as a random effect, and all variables were standardized prior to analyses. We specified uninformative priors with normal priors (mean = 0, SD = 10) for coefficient estimates and cauchy prior (scale = 2) for standard deviation. We checked for co-linearity between independent variables using pair plots and Variance Inflation Factors (VIF) for all explanatory variables. We considered there to be no collinearity in the variables when all VIF’s were less than 3.0^42^. The model was analyzed using Markov Chain Monte Carlo methods implemented in Stan, run from R 3.6.3 (R Development Core Team, 2016) using the package BRMS^43^. We ran 4 chains of 4,000 iterations, with a warm-up of 500 iterations. We assessed convergence visually with history plots and with the Rˆ statistic; no evidence of lack of convergence existed (maximum Rˆ < 1.01). Unless otherwise indicated, we report the posterior median and 95% credible interval. We report all methods and results in accordance with the Arrive Guidelines.

## Supplementary Information

Supplementary Information 1.

Supplementary Information 2.

## Data Availability

Data and code are provided in Supplement 1.
